# Whole Transcriptome Analysis Reveals a Potential Regulatory Mechanism of LncRNA-FNIP2/miR-24-3p/*FNIP2* Axis in Chicken Adipogenesis

**DOI:** 10.3389/fcell.2021.653798

**Published:** 2021-06-24

**Authors:** Lijin Guo, Xiaohuan Chao, Weiling Huang, Zhenhui Li, Kang Luan, Mao Ye, Siyu Zhang, Manqing Liu, Hongmei Li, Wen Luo, Qinghua Nie, Xiquan Zhang, Qingbin Luo

**Affiliations:** ^1^Guangdong Laboratory, Lingnan Modern Agricultural Science and Technology, South China Agricultural University, Guangzhou, China; ^2^College of Animal Science, South China Agricultural University, Guangzhou, China; ^3^School of Biological and Chemical Sciences, Queen Mary University of London, London, United Kingdom

**Keywords:** whole transcriptome analysis, lncRNA, miRNA, mRNA, lipid synthesis, adipogenesis

## Abstract

Lipid biosynthesis is a complex process, which is regulated by multiple factors including lncRNA. However, the role of lncRNA in chicken abdominal fat accumulation is still unclear. In this research, we collected liver tissues from six high abdominal fat rate Sanhuang broilers and six low abdominal fat rate Sanhuang broilers to perform lncRNA sequencing and small RNA sequencing. A total of 2,265 lncRNAs, 245 miRNAs, and 5,315 mRNAs were differently expressed. Among of them, 1,136 differently expressed genes were enriched in the metabolic process. A total of 36 differently expressed genes, which were considered as differently expressed lncRNAs’ targets, were enriched in the metabolic process. In addition, we also found out that eight differently expressed miRNAs could target 19 differently expressed genes. *FNIP2* and *PEX5L* were shared in a cis-regulatory network and a differently expressed miRNA target relationship network. LncRNA-FNIP2/miR-24-3p/*FNIP2* axis was considered as a potential candidate that may participate in lipid synthesis. Experimentally, the objective reality of lncRNA-FNIP2/miR-24-3p/*FNIP2* axis was clarified and the regulation effect of lncRNA-FNIP2/miR-24-3p/*FNIP2* axis on synthesis was validated. In brief, our study reveals a potential novel regulatory mechanism that lncRNA-FNIP2/miR-24-3p/*FNIP2* axis was considered as being involved in lipid synthesis during chicken adipogenesis in liver.

## Background

Adipose tissue is a signal hub that regulates systemic metabolism through paracrine and endocrine signals. The liver is an important organ for the synthesis and accumulation of fat and has a regulatory role in lipid metabolism (Ma et al., 2018). During the past few decades, intensive genetic selection breeding in commercial broilers has gained satisfactory achievements in increased growth rates and egg production, but also led to undesirable fat deposition (Liang et al., 2015; Resnyk et al., 2017). Excessive fat deposition in broilers has thus become a major problem in today’s broiler industry, with the reason that it causes a reduction on the carcass yield and feed efficiency, and loss of consumers’ favor for the meat as well (Wang et al., 2017). It has been proven that fat deposition is regulated by a variety of signal pathways and gene networks, among which, non-coding RNAs are the main regulators (Alvarez-Dominguez et al., 2015; Hootman et al., 2017; Zheng et al., 2017). A better understanding of the mechanism underlying adipogenesis in chicken will bring benefits to the sustainable development of poultry industry. Whole-transcriptome sequencing has enabled the identification and characterization of a variety of putative relevant non-coding RNAs (Wang et al., 2015; Zhang et al., 2017a).

Long non-coding RNAs (lncRNAs) are a class of non-coding RNA with a length longer than 200 nucleotides (Quinn and Chang, 2016; Ransohoff et al., 2018). MicroRNAs (miRNAs) are a class of small non-coding RNA of approximately 22 nucleotides in length (Krol et al., 2010; Lu and Rothenberg, 2018). It is well established that lncRNA can act as a miRNA sponge through competing endogenous RNA (ceRNA) activity, thereby regulating the gene expression of miRNA (Tay et al., 2014; Zhang et al., 2018). Fat acid could be synthesized by hepatic *de novo* lipogenesis (DNL) from carbohydrates (especially glucose) and the liver is the main site for fatty acid metabolism and triglyceride synthesis in vertebrates (Hudgins et al., 1996; Chakravarthy et al., 2005; Chang et al., 2006; Chakravarthy et al., 2009; Jensen-Urstad and Semenkovich, 2012), but the regulatory mechanism of adipogenesis in chicken liver is still unclear. Investigating the RNA interaction will shed light on the gene regulatory network in the progression of chicken adipogenesis. To achieve this goal, we carried out an extensive profiling of transcriptome in the liver tissues from high-fat and low-fat chickens by RNA-Seq, screening out the differently expressed lncRNA (DEL), miRNA (DES), and mRNA (DEM).

In this study, we obtained 2,265 DELs, 245 DESs, and 5,315 mRNAs, many of which were involved in lipid metabolism. In addition, we identified multiple candidate genes and regulatory networks, including the lncRNA-FNIP2/miR-24-3p/*FNIP2* axis, which was considered as being involved in lipid biosynthesis. In total, our data not only gained novel insights into regulatory mechanism in chicken adipogenesis but also contributed to a better understanding of epigenetics.

## Materials and Methods

### Ethics Statement

All animals used in this research were sourced from Wens. All animal experiments were conducted with the guarantee that the animals suffer minimal pain. The animal experiments were approved by the Animal Care Committee of South China Agricultural University (Approval ID: SCAU#2017015; September 13, 2017).

### Samples for RNA Sequencing

After birth, 12 broilers (Sanghuang chicken) were placed in a cage with free feeding. At the 100th day, the 12 broilers were euthanized by cervical dislocation. The abdominal fat tissues were isolated and weighed. According to their abdominal fat ratio, they were divided into two groups, high-fat group and low-fat group. The abdominal fat ratio in the low-fat group ranged from 2.81 to 3.89%. The abdominal fat ratio in the high-fat group ranged from 8.04 to 9.75%. The liver tissues from the two groups were collected for RNA sequencing.

### Total RNA Extraction, cDNA Library Construction, and RNA Sequencing

Total RNA was extracted by using the Trizol Reagent (Invitrogen, Carlsbad, CA, United States) following its manufacturer’s protocol. RNA integrity was detected in agarose gel electrophoresis and RNA concentration and purity were determined by the NanoDrop 2100 (Thermo Fisher Scientific, Fremont, CA, United States). Ribo-Zero^TM^ rRNA Removal Kit (Epicentre, Madison, Wisconsin, United States) was used to remove the ribosomal RNA (rRNA). After purification, the RNA was fragmented. Subsequently, cDNA first strand was synthesized by using the TruSeq^®^ Stranded kit (Illumina, San Diego, CA, United States) according its protocol. DNase I (Thermo Fisher Scientific, Fremont, CA, United States) and RNase H (Thermo Fisher Scientific, Fremont, CA, United States) were used in the synthesis of the second strand of cDNA. The poly A tail and adapter were attached into the double-strand cDNA. After amplification and purification, cDNA library was obtained for RNA sequencing. BGI was responsible for RNA sequencing. The raw data obtained from sequencing were filtered to obtain clean data. The clean data was mapped to the reference genome (GRCg6a) by using HISAT (Kim et al., 2015). StringTie (Pertea et al., 2015) was used in the transcript assembly. For novel transcripts, they were divided into mRNA and lncRNA according to their coding ability, which were predicted by the CPC (Kong et al., 2007), txCdsPredict (Sun et al., 2013), CNC (Sun et al., 2013), and pfam database (Finn et al., 2016). CPC score < 0, txCdsPredict score < 500, CNC score < 0, and out of pfam database were considered as the evaluation standard for lncRNA. When at least three of the four methods were consistent, the transcript could be identified as an mRNA or lncRNA. The clean reads were aligned to reference sequences by using Bowtie2 (Langmead and Salzberg, 2012). The expression of genes or transcripts was calculated by RSEM (Li and Dewey, 2011). The filtering conditions for the significant differently expressed transcripts were set as |Fold Change| ≥ 2 and *Q*-value ≤ 0.001. Cluster analysis for differently expressed transcripts was performed by pheatmap 1.0.8. The raw data obtained from RNA sequencing was submitted in the SRA database (accession link^1^).

### Function Annotation of mRNA

The mRNA (including novel and known mRNA) was annotated in the NR and KEGG databases. Blast 2.2.23 (Altschul et al., 1990) and Diamond 0.8.31 (Buchfink et al., 2015) were used in the mRNA NR and KEGG annotation. Blast2GO 2.5.0 (Conesa et al., 2005) and NR annotation were used in the Gene Ontology (GO) annotation. The parameters used in the annotation were all default parameters.

### LncRNA Target Gene Prediction and *Cis*-regulated Networks Construction

*Cis*-regulation or trans regulation on target genes were considered as the ways for lncRNA function. For cis regulation, lncRNA function was associated with its adjacent coding gene which could be regarded as its target gene. If lncRNA was located within the upstream 10 kb or downstream 20 kb from its adjacent mRNA, it would be judged as a cis regulation. If the lncRNA was out of the range and MEF (between lncRNA and mRNA) < -30 kcal/mol, it would be judged as a trans regulation. The Spearman correlation ≥ 0.6 and Pearson correlation ≥ 0.6 between lncRNA and mRNA were taken as the criteria for their target relationship. The intersection of differentially expressed mRNA and predicted target mRNA was selected to construct the cis-regulated networks between DELs and potential targets. Cytoscape 3.7.2 was used to construct the cis-regulated networks.

### Small RNA Library Construction and Sequencing

The RNA sized 18–30 nt was isolated from total RNA by agarose gel electrophoresis, then their 3′ region with 5-adenylated and 3-blocked single strand DNA adapter were ligated. The RNA 5′ region was ligated with another adapter. The RT primer with Unique Molecular Index (UMI), hybridized with the 3′ adapter, was used to synthesize the first strand of cDNA. After amplification, the PCR productions sized 100–120 bp were isolated for quality examination. The qualified library was used in small RNA sequencing. The raw data obtained from small RNA sequencing were submitted on the SRA database (accession link^2^). After removing the none-insertion fragment sequences, long-insertion fragments sequences, low-quality sequences, polyA sequences, and small-fragment sequences, the clean data was mapped to the reference genome (GRCg6a) by using the AASRA software (Chong et al., 2017). All unique RNA was sorted into a different kind RNA by annotating in the small RNA database. The priority for annotation was MiRbase > pirnabank > snoRNA > Rfam. Novel miRNAs were predicted by using miRDeep2^3^ (Friedl Nder et al., 2008). DEGseq^4^ (Wang et al., 2010) was used to analyze the differentially expressed miRNA. Significantly differentially expressed miRNAs with |Fold Change| ≥ 2 and *Q*-value ≤ 0.001 were considered.

### Mirna Target Gene Prediction and DES-DEM Target Network Construction

Target gene prediction was performed by using miRanda (accession link^5^) (John et al., 2004) and RNAhybrid (accession link^6^) (Kruger and Rehmsmeier, 2006). For the identification of potential target genes, RNAhybrid ≤ -30 kcal/mol, MEF miRanda MEF ≤ -45 kcal/mol, and miRanda score ≥ 300 were considered as the filter criteria. These selected potential targets were compared with the DEMs in the above RNA sequencing and differently expressed potential targets were selected for DES-DEM target network construction. Cytoscape 3.7.2 was used for network construction.

### Reverse Transcription Reaction and Quantified Real Time PCR (qRT-PCR)

Reverse transcription reaction was performed by using the HiScript^®^ II Q RT SuperMix for qPCR (+gDNA wiper) (Vazyme, Nanjing, Jiangsu, China) and miRNA 1st Strand cDNA Synthesis Kit (by stem-loop) (Vazyme, Nanjing, Jiangsu, China) following their manufacturers’ protocol, respectively. Quantified real time PCR (qRT-PCR) was carried out in the ABI QuantStudio 5 instrument (Thermo Fisher Scientific, Fremont, NY, United States) by using the ChamQ Universal SYBR qPCR Master Mix (Vazyme, Nanjing, Jiangsu, China). U6 and GAPDH were respectively considered as the reference genes in qRT-PCR for miRNA, mRNA, and lncRNA expressions. The primers used in miRNA qRT-PCR were designed and synthesized by RioBio (Guangzhou, Guangdong, China). The primers used in lncRNA and mRNA qRT-PCR were designed by the Premier Primer 5.0 software and synthesized by TSINGKE Biotech (Guangzhou, Guangdong, China). The primers we designed were listed in Supplementary Table 1.

### Oligonucleotides Synthesis and Plasmid Construction

The oligonucleotides used in this study (including miR-24-3p mimic and miR-24-3p inhibitor) were synthesized by RioBio (Guangzhou, Guangdong, China) and they were listed in Supplementary Table 1. The wild type sequences and mutated type sequences of lncRNA-FNIP2 and FNIP2 3′UTR with miR-24-3p binding sites were synthesized and cloned into pmiR-GLO vector by TSINGKE Biotech (Guangzhou, Guangdong, China). The mutated type sequences were transformed from wild type sequences with the binding sites which mutated from “CUGAGCU” to “GCACAUC” or from “CUGAGCU” to “GCACAUC.”

### Cell Culture and Cell Transfection

Immortalized chicken preadipocytes 1 (ICP1) was provided by the Hui Li research team from Northeast Agricultural University. ICP1 was cultured in DMEM/F12 medium (Gibco, Grand Island, NY, United States) with 10% fetal bovine serum (Gibco, Grand Island, NY, United States) and 0.1%/0.2% penicillin/streptomycin (Gibco, Grand Island, NY, United States) in an incubator with 5% CO2 at 37°C. Lipofectamine 3000 reagent (Invitrogen, Carlsbad, CA, United States) was used to perform cell transfection by following its manufacturer’s protocol. The transfection dose of DNA was 1 μg/well or 0.1 μg/well for a 12-well plate or a 96-well plate, respectively. For oligonucleotides, the final transfection concentration was 50 μM.

### Dual-Luciferase Reporter Assay

Wild sequences (WT) and mutational sequences (MT) of lnc-FNIP2 and *FNIP2* 3′UTR were respectively cloned into the pmiR-GLO vector. The transfection treatment combinations in the dual-luciferase reporter assay were respectively “WT + mimic,” “MT + mimic,” “WT + mimic NC,” and “MT + mimic NC.” Dual-luciferase reporter assay was performed by using a dual-luciferase reporter assay kit (Vazyme, Nanjing, Jiangsu, China) by following its manufacturer’s protocol. The firefly luciferase and Renilla luminescence activities were measured in a multi-function microplate reader (Biotek, Winooski, VT, United States).

### Oil Red O Staining

The ICP1 cells were induced to differentiate by using the culture medium with 15% fetal serum and 0.2% oleic acid (Sigma, St Louis, CA, United States) after 12-h transfection. After 48-h induction, oil red O staining was performed by using an Oil Red Staining kit (Solarbio, Beijing, China) according to its protocol. The stained cells were captured in an electric microscope (Nikon, Tokyo, Japan). The oil red dye was extracted from the cells by using isopropanol solution. At last, the dye was quantified by a microreader (Biotek, Winooski, VT, United States).

### Western Blot

The ice-cold radio immunoprecipitation (RIPA) lysis buffer (Beyotime, Shanghai, China) with 1 mM phenylmethyl sulfonyl fluoride (Beyotime, Shanghai, China) was used in the total protein extracted from liver tissues or ICP1 cells. 10% SDS-page gel was used to separate proteins and the separated proteins were transferred to the polyvinylidene fluoride (PVDF) membrane (Bio-Rad, Hercules, CA, United States). After 30-min of blocking, the membrane was incubated with anti-PPARγ (1:1,000; bs-0530R, BIOSS), anti-FNIP2 (1:500; bs-13194R, BIOSS), or anti-GAPDH (1:2,000; bsm-33033M, BIOSS) at 4°C for 12 h. Anti-mouse secondary antibody (1:10,000; 7076P2, CST) or anti-rabbit secondary antibody (1:10,000; 7074P2, CST) was used to incubate the membranes. ECL Peroxidase Color Development Kit (Vazyme, Nanjing, Jiangsu, China) was used in chromogenic reaction by following the manufacturer’s protocol. The protein bands visualization was performed in the Odyssey instrument (Li-cor, Lincoln, NE, United States).

### Data Analysis

The data was represented as mean ± SEM. The statistical significance of differences between groups were evaluated by the Student’s *t*-test. ^∗^*P* < 0.05; ^∗∗^*P* < 0.01; ^∗∗∗^*P* < 0.001; ^****^P < 0.0001; ns, no significance.

## Results

### Blast Analysis of Transcriptome Sequencing

According to abdominal fat ratio, twelve 100-day-old Sanhuang broilers were divided in high-fat and low-fat groups. The liver tissues from the 12 broilers were collected for whole transcriptome sequencing. We obtained 12.86 Gb of raw data for each sample on average (Supplementary Table 2) and they were submitted to the NCBI database (accession ID: PRJNA684949). After filtrating the raw reads, 12.54 Gb of total clean reads were obtained. The average GC content reached 47.05% and the base proportion reaching Q30 standard was not less than 92.41%. The clean reads were mapped to the reference genome (GRCg6a), average mapping ratio was 89.57%, and unique mapping ratio was not less than 83.08% (Supplementary Table 3).

In order to distinguish mRNA and lncRNA, the coding capacity of transcripts was predicted by three softwares and the pfam database. In this sequencing, a total of 52,306 transcripts were detected and 25,760 of them were novel transcripts, including 9,861 novel lncRNAs (Supplementary Figure 1a), 12,978 novel mRNAs (Supplementary Figure 1b), 5,106 known lncRNAs, and 25,433 known mRNAs. Here, we showed the distribution of RNA length (including the novel and the known). The main length distribution of lncRNA is from 500 to 1,500 bp, while the mRNA is from 1,000 to 3,000 bp (Supplementary Figure 1c). Bowtie2 was used to align clean reads to the reference sequences, and RESM was then used to calculate gene and transcript expression. The number statistics of isoforms (lncRNA and mRNA) for 12 samples was listed in Supplementary Table 4. In order to visualize the number of genes in each sample in different FPKM intervals, we counted the number of genes in three conditions of FPKM (FPKM ≤ 1, 1 < FPKM < 10, FPKM ≥ 10). The number of transcripts which FPKM ≥ 10 was not less than 3,206 (Supplementary Figure 1d).

### DEG Between Liver Tissues From High-Fat to Low-Fat Chickens

**FIGURE 1:**
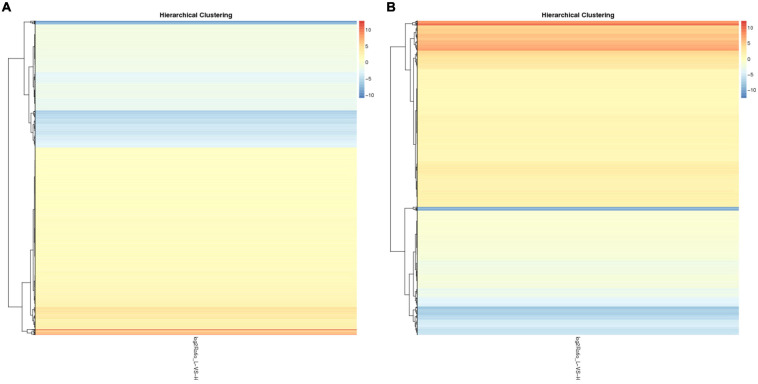
The cluster of DELs and DEMs. **(A)** The cluster of 2,265 DELs by the pheatmap software. **(B)** The cluster of 5,315 DEMs by the pheatmap software.

**FIGURE 2:**
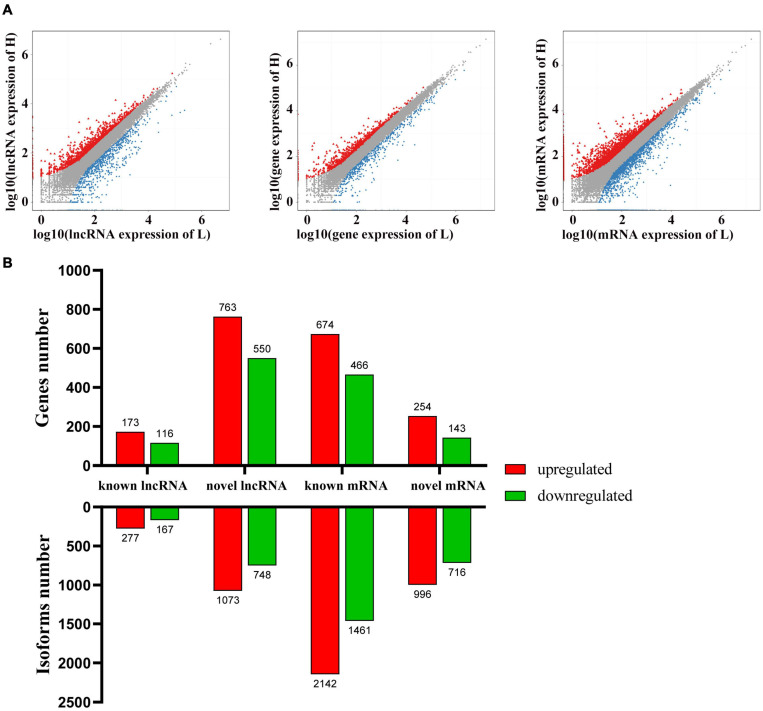
Differently expressed gene between liver tissues from high-fat and low-fat chickens. **(A)** Scatter plots of lncRNA, gene and mRNA expression distribution. Color: Blue indicates down-regulated genes, red indicates up-regulated genes, and gray indicates non-differently expressed genes. **(B)** The number of differently expressed lncRNAs and mRNAs at the gene and isoform levels.

To identify the potential candidate genes related to adipogenesis, the expression levels of lncRNA and mRNA were examined in liver tissues from high-fat and low-fat chickens. *Q*-value ≤ 0.001 and | fold change| ≥ 2 were set as the standard for differential expression. A total of 2,265 lncRNAs (Supplementary Table 5) and 5,315 mRNAs (Supplementary Table 7) were differently expressed between liver tissues in high-fat and low-fat chickens. The differently expressed lncRNAs (DELs) and differently expressed mRNAs (DEMs) were clustered (Figures 1A,B) by the pheatmap software, according to their fold change. The number of DELs (Supplementary Table 5), differently expressed genes (DEGs) (Supplementary Table 6), and DEMs (Supplementary Table 7) between the different groups are shown in Figure 2A. In these 2,265 DELs, including 1,821 novel lncRNAs and 444 known lncRNAs, 1,350 lncRNAs were upregulated and 915 lncRNAs were downregulated. Among the 5,315 DEMs, 3,138 and 2,177 mRNAs were upregulated and downregulated, respectively. Besides, we made statistics on the differentially expressed genes at the isoform and gene levels, respectively (Figure 2B). A total of 1,864 DEGs were upregulated and 1,275 DEGs were downregulated.

The top 10 abundant DELs and top 10 abundant DEMs were listed in Tables 1 and 2, respectively. All the top 10 abundant DELs were novel lncRNAs and only two of them were upregulated. LTCONS_00020831 (log2FC = 1.21, *Q*-value = 0), the most abundant DEL in this sequencing, was upregulated in liver tissues from high-fat chickens. Eight of the top 10 abundant DEMs were downregulated, and seven of the top 10 abundant DEMs were known transcripts. For DEMs, ENSGALT00000002892 (*VTG2*, vitellogenin 2, log2FC = –1.73992, *Q*-value = 0) was highly expressed in the liver of low-fat chickens and showed the highest abundance in DEMs.

**TABLE 1:** The top 10 abundant DELs between the high-fat and low-fat groups.

LncRNA ID	Length	L Reads number	H Reads number	log2FC(H/L)	H/L	*q*-Value
LTCONS_00020831	1,186	79,374.24	168,343.7	1.213923	Up	0
LTCONS_00020832	1,351	230,312.4	5,371.91	–5.29276	Down	0
LTCONS_00055244	43,793	113,243	49,591	–1.06202	Down	0
LTCONS_00029013	512	158,164.8	4,411.57	–5.03474	Down	0
LTCONS_00040763	2,932	73,239.73	30,631.24	–1.12837	Down	0
LTCONS_00046846	24,766	77,193.92	3,376.17	–4.38577	Down	0
LTCONS_00000249	10,649	50,129.79	19,380	–1.24184	Down	0
LTCONS_00025768	41,916	47,501.63	20,827.96	–1.0602	Down	0
LTCONS_00010452	68,068	21,954.88	40,581.95	1.015552	Up	0
LTCONS_00000621	12,396	45,945.24	12,643.59	–1.73225	Down	0

**TABLE 2:** The top 10 abundant DEMs between the high-fat and low-fat groups.

mRNA ID	Length	L Reads number	H Reads number	log2FC (H/L)	H/L	*q*-Value
ENSGALT00000002892	5,565	2,165,799	592,843.8	–1.73992	Down	0
ENSGALT00000056823	5,951	959,658	234,419	–1.90418	Down	0
MTCONS_00058597	6,098	282,406.2	119,824.5	–1.10759	Down	0
ENSGALT00000083486	1,927	371,304	112,513	–1.59325	Down	0
ENSGALT00000027531	4,885	24,398.34	84,566.61	1.922561	Up	0
ENSGALT00000019639	1,603	168,144	70,319	–1.12846	Down	0
ENSGALT00000068577	805	26,347	59,925.02	1.314775	Up	0
MTCONS_00056041	5,126	545,124.6	59,193.44	–3.07382	Down	0
ENSGALT00000024496	2,764	159,362	58,913	–1.30639	Down	0
MTCONS_00043232	2,325	132,570.9	58,860.85	–1.04213	Down	0

### Functional Enrichment Analysis of DEMs Involved in Lipid Metabolism

**FIGURE 3:**
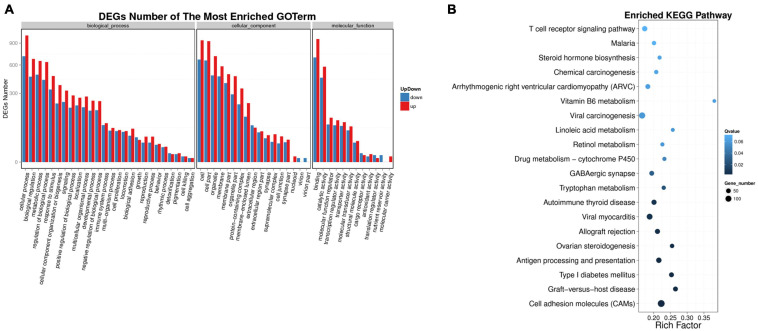
Functional enrichment analysis of DEGs. **(A)** DEGs number of the most enriched GO term. *X*-axis: GO terms; *y*-axis: gene number; blue bar: downregulated DEG; red bar: upregulated DEG. **(B)** The top 20 KEGG pathways DEGs enriched in. *X*-axis: rich factor; *y*-axis: pathway.

In order to explore the functions of DEMs involved in chicken lipid metabolism, GO enrichment analysis was performed in this study (Supplementary Table 8). Here, we only analyze the DEGs corresponding to DEMs. As shown in Figure 3A, 1,136 DEGs were enriched in the metabolic process (650 upregulated and 486 downregulated DEGs), which may be associated with chicken abdominal fat deposition. In addition, 65 upregulated and 61 downregulated DEGs were enriched in cell proliferation. To determine in which pathways the DEGs between high-fat and low-fat chickens are more concentrated, Kyoto Encyclopedia of Genes and Genomes (KEGG) enrichment analysis was performed. *Q*-value < 1 was set as the cut off for the significantly enriched pathways. The top 20 enriched KEGG pathways were shown in Figure 3B. Our result suggested that the DEGs were significantly enriched in *Cell adhesion molecules* (*CAMs*), *Graft-versus-host disease*, *Type I diabetes mellitus*, and so on. Here, we showed all the DEGs enriched pathways (Supplementary Table 9). A total of 494 DEGs were significantly enriched in the *Metabolic pathways*, which may be involved with lipid metabolism during chicken abdominal fat accumulation.

### Target Gene Prediction of DEL

**FIGURE 4:**
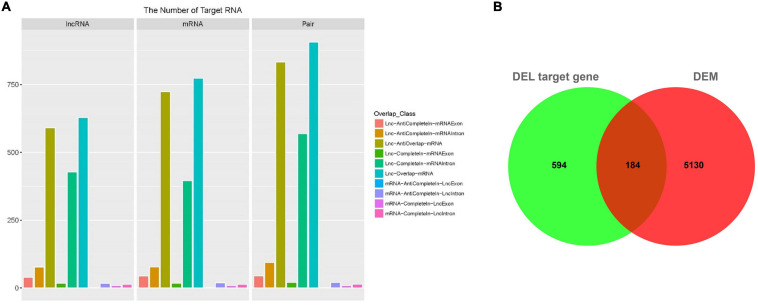
Target gene prediction of DEL. **(A)** The overlap classification between differently expressed lncRNAs and predicted target mRNA. *X*-axis: lncRNA, mRNA, and pair. *Y*-axis: number. Color: different kind of overlap which is shown in the right side of the figure. **(B)** Venn diagram of the intersection between the DEL target gene and differently expressed mRNAs.

The function of lncRNA is mainly achieved by acting on the target genes in cis mode. The basic principle of cis target gene prediction is that the function of lncRNA is related to the protein coding genes adjacent to its coordinates, so the mRNA adjacent to lncRNA is screened out as its target gene. Here, we totally obtained 9,242 target pairs between lncRNA and their adjacent mRNA (Supplementary Table 10). A total of 2,634 of these target pairs were localized at the 10K range upstream of the mRNAs, and the target pair number of the distance between the upstream lncRNA and the mRNA ranging from 1 to 1,000 nt was 796. On the other hand, 4,003 target pairs were localized at the 20K range downstream of the mRNAs, and the distance of 513 pairs were within 1,000 nt from downstream lncRNA to mRNA. In addition, there was an overlap between 1,820 lncRNA and 2,075 mRNA, and 2,512 target pairs were formed (Figure 4A and Supplementary Table 11). There were 628 lncRNAs that overlapped with 774 mRNAs, forming 907 target pairs. In addition, 591 lncRNAs anti-overlapped with 724 mRNAs forming 833 target pairs. Spearman-correlation ≥ 0.6 and Pearson-correlation ≥ 0.6 between lncRNA and mRNA were considered as the cut off for DELs’ target genes, and we obtained 778 DELs’ target genes. The 778 target genes were compared with DEMs, 184 mRNAs of which were differently expressed (Figure 4B).

### Functional Enrichment Analysis of Differently Expressed DELs’ Target Genes

**FIGURE 5:**
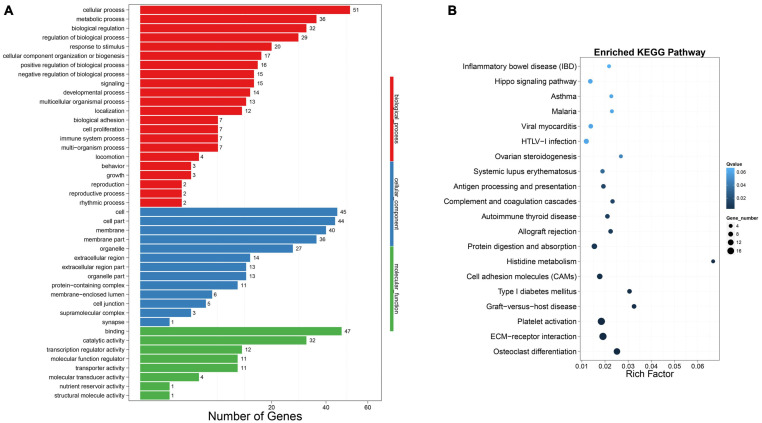
Functional enrichment analysis of DELs’ target genes. **(A)** Gene number of the most enriched GO term. **(B)** The top 20 KEGG pathways DELs’ targets enriched in.

To evaluate the biological function of these differently expressed DELs’ target genes, we carried out the functional enrichment analysis. GO function enrichment analysis showed that 36 DEMs were enriched in the metabolic process (Figure 5A). The 36 DEMs might participate in lipid metabolism and chicken abdominal fat deposition, regulated by lncRNAs to achieve this biological process. In addition, there were 29, 14, and seven DEMs, respectively, enriched in the regulation of biological process, developmental process, and cell proliferation, which may be involved in abdominal fat adipocytes proliferation and differentiation. KEGG enrichment analysis was also performed to determine which pathways were more concentrated in which target genes were significantly different between groups. *Q*-value < 0.05 was considered as the criteria for the significant differences and the DEMs could be enriched in 142 pathways (Supplementary Table 12). Here, we showed the top 20 significantly enriched pathways in Figure 5B. The most enriched pathways are the *Osteoclast differentiation*, *ECM-receptor interaction*, *Platelet activation, Graft-versus-host disease*, and *Type I diabetes mellitus*. The 23 DEMs, enriched in the *Metabolic pathways*, were considered to associate with the liver regulation of abdominal fat deposition (Supplementary Table 12).

### DEL-DEG Interaction Network Construction

**FIGURE 6:**
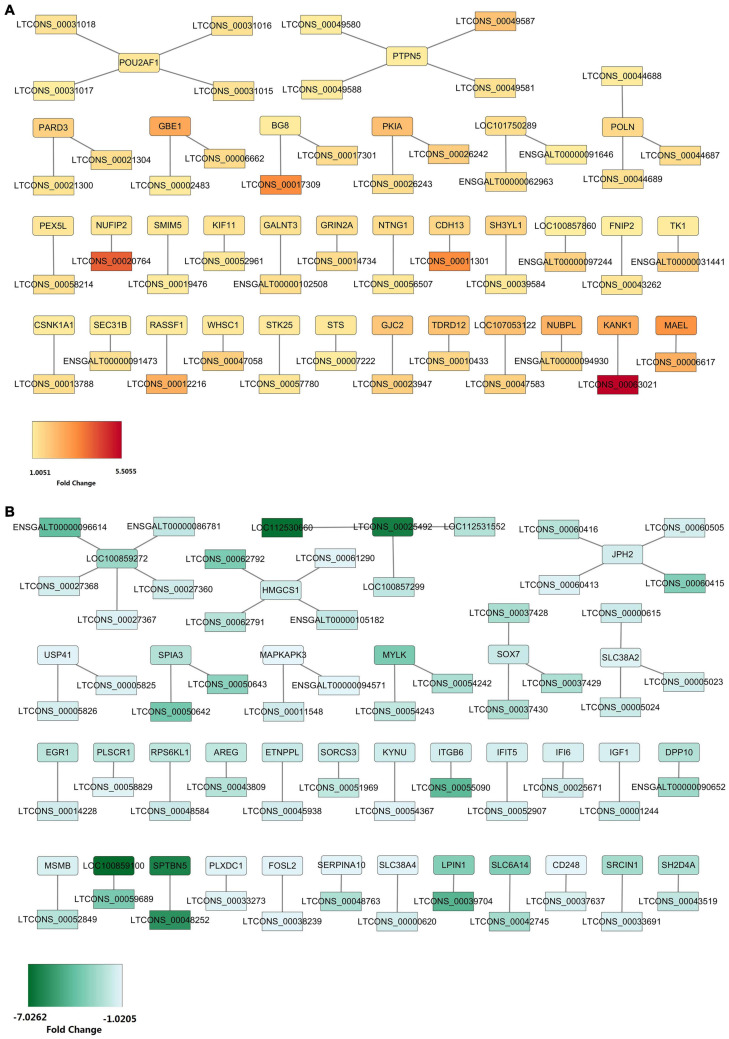
DEL-DEG interaction network construction. **(A)** The interaction network between 45 upregulated DELs and 32 upregulated DEGs. **(B)** The interaction network between 52 downregulated DELs and 36 downregulated DEGs.

To further evaluate how DELs come into play in regulating their targets, we carried out the DEL-DEG interaction network construction. Here, we found that the 184 DEMs, which we obtained above, were transcribed from 68 genes and we obtained 99 target pairs between the 68 DEGs and 97 DELs (Supplementary Table 13). We constructed two target interaction networks between the upregulated DELs and upregulated DEGs or downregulated DELs and downregulated DEGs, respectively (Figure 6). A total of 45 target pairs were formed by 45 upregulated DELs and 32 upregulated DEGs (Figure 6A). In this network, lncRNA-GBE1 (LTCONS_00002483, log2FC = 1.07), lncRNA-PEX5L (LTCONS_00058214, log2FC = 1.34), lncRNA-PARD3 (LTCONS_00021300, log2FC = 1.22), lncRNA-NTNG1 (LTCONS_00056507, log2FC = 1.14), and lncRNA-FNIP2 (LTCONS_00043262, log2FC = 1.20) were the top five abundant lncRNAs, which could respectively target to *GBE1* (log2FC = 2.60), *PEX5L* (log2FC = 1.20), *PARD3* (log2FC = 1.67), *NTNG1* (log2FC = 1.44), and *FNIP2* (log2FC = 1.01), indicating their potential cis regulatory relationship. On the other hand, 54 target pairs were formed by 52 downregulated DELs and 36 downregulated DEGs (Figure 6B). In the downregulated DEL-downregulated DEG interaction network, the top five abundant lncRNAs were lncRNA-SPIA3 (LTCONS_00050642, log2FC = –3.71), lncRNA-SLC38A2 (LTCONS_00005023, log2FC = –1.47), lncRNA-IGF1 (LTCONS_00001244, log2FC = –1.63), lncRNA-JPH2 (LTCONS_00060413, log2FC = –1.25), and lncRNA-SOX7 (LTCONS_00037430, log2FC = –2.29). In addition, in our previous sequencing, *LPIN1* was downregulated in the abdominal fat tissue from the high-fat Sanhuang chicken, which, in this sequence, its downregulation was also found. This network revealed a novel potential regulatory mechanism that a low expression of lncRNA-LPIN1 (LTCONS_00039704) mediates the down-regulation of *LPIN1* expression and, thus, affects lipid metabolism.

### MiRNA Expression Profile

**FIGURE 7:**
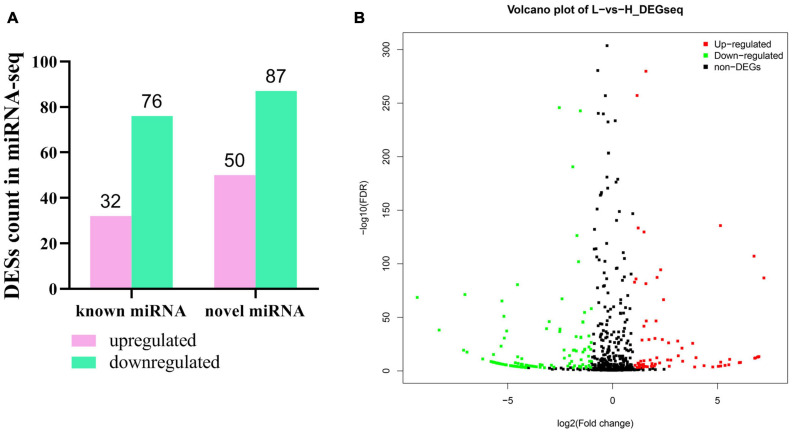
Differently expressed miRNA in liver between high-fat and low-fat chickens. **(A)** Statistic of differently expressed miRNA in liver between high-fat chicken and low-fat chickens. **(B)** The volcano plot of differently expressed miRNA in liver between high-fat chicken and low-fat chickens.

In order to further clarify the potential regulatory mechanism of abdominal fat deposition in an epigenetic perspective, the RNA, used in lncRNA sequencing, was used in a small RNA sequencing on the BG1SEQ-500 platform. In total, we obtained an average of 24.24 Gb of raw data for each sample and the raw data were submitted to the SRA database (accession ID: PRJNA686699). After filtering, an average of 21.73 Gb of clean data for each sample was obtained and the percentage of clean tag reached 89.63% (Supplementary Table 14). The clean tag was mapped to the known small RNA databases, including miRBase, Rfam, siRNA, piRNA, and snoRNA, and the mapped rate ranged from 83.62 to 94.25% (Supplementary Table 14). In this sequencing, we detected 1,100 microRNAs (miRNAs), including 545 known miRNAs and 555 novel miRNAs. | Fold change| ≥ 2 and *q*-Value ≤ 0.001 were considered as a significant difference of miRNA expression. In total, 245 DESs were found, including 137 novel miRNAs and 108 known miRNAs (Figure 7A). Among them, 82 miRNAs were upregulated and 163 miRNAs were downregulated (Figure 7B). We listed the top 20 abundant differently expressed miRNAs in Table 3. There were nine and 11 miRNAs that were upregulated and downregulated, respectively. The most abundant DES was miR-92a_1 (FC = -1.78953), which was downregulated in the liver of chickens with a high abdominal fat rate.

**TABLE 3:** The top 20 abundant differently expressed miRNAs.

miRNA id	Expression (L)	Expression (H)	log2Ratio (H/L)	Up/down	*q*-Value
miR-92a_1	35,074	7,418	–1.78953	DOWN	0
miR-191-5p	27,280.33	9,062.833	–1.13806	DOWN	0
miR-100-5p_1	10,997.17	24,209	1.590181	UP	0
miR-126-3p	22,141	2,196.833	–2.88145	DOWN	0
miR-26c_1	412.5	21,795.17	6.17524	UP	0
miR-126-3p_1	2,961	17,333.33	3.001163	UP	0
miR-92a_2	1,384.167	17,077	4.076735	UP	0
novel_mir182	7,226.5	303.3333	–4.12255	DOWN	0
miR-429	2,103	4,070.5	1.404527	UP	0
miR-125b_1	2,115.667	3,120.167	1.012281	UP	0
miR-10a-5p	4,031	995	–1.5666	DOWN	0
miR-24-3p	3,729.667	1,236.5	–1.14101	DOWN	0
novel_mir4	3,346.167	1,189.333	–1.04059	DOWN	0
let-7b	3,158.333	1,033.833	–1.15939	DOWN	0
miR-146a_1	3,093.333	716.6667	–1.65802	DOWN	0
miR-140-5p_1	1,253.667	2,049.5	1.160888	UP	0
miR-146a-5p_1	1,103.667	2,170.167	1.427271	UP	0
miR-429_1	3,218.167	42.16667	–5.80222	DOWN	0
miR-10a_2	638.8333	2,542.5	2.444506	UP	0
miR-103_1	1,747.5	602	–1.08569	DOWN	0

### The Target Prediction of Differently Expressed miRNAs

**FIGURE 8:**
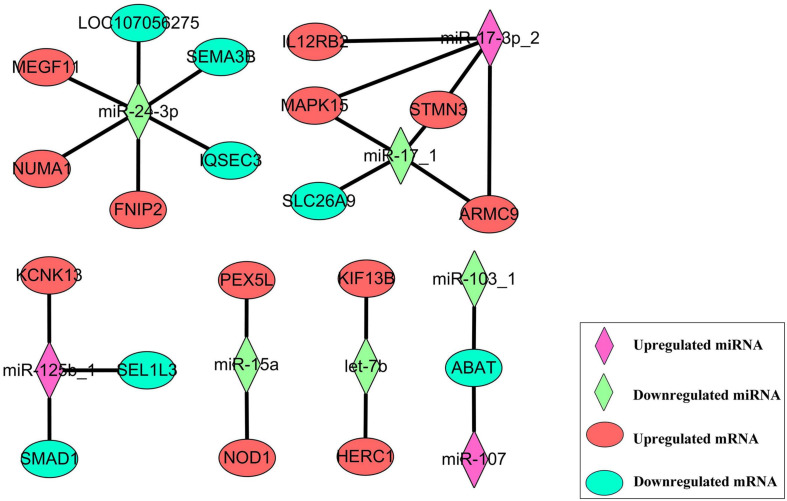
The target relationship network between eight DESs and 19 DEGs.

To evaluate the regulatory mechanism of these differently expressed miRNAs, target prediction was performed by RNAhybrid and miRanda. The filter parameters of screening potential target mRNA were as follows: RNAhybrid MEF ≤ -30 kcal/mol, miRanda MEF ≤ -45 kcal/mol, and miRanda score ≥ 300. In total, 1,407 target pairs were formed by 32 known miRNAs and 1,068 mRNAs. These potential mRNAs were compared to the DEGs we obtained in the above whole transcriptome sequencing, and we found 23 target pairs between 19 DEGs and 8 DESs, including 13 negative target pairs and 10 positive target pairs. Based on the circumstances of regulation on mRNA caused by miRNA, we performed the target relationship network construction (Figure 8). In this network, we could find that miR-24-3p was at a critical node position, interacted with six DEGs, including three negative interaction and three positive interaction. miR-17-3p_2 had four positive targets, three of them were negatively regulated by miR-17_1.

The regulatory networks between DELs and DEGs were compared with the regulatory network between DESs and DEGs, in which *FNIP2* and *PEX5L* was found out to be common to both networks. The low expression of miR-15a may be responsible for the upregulation of *PEX5L* which may also be cis regulated by lncRNA-PEX5L. Similar in *PEX5L*, the upregulation of *FNIP2* may be caused by miR-24-3p low expression and lncRNA-FNIP2 high expression. Interestingly, it was found that lncRNA-FNIP2 has the potential to target and bind miR-24-3p (Supplementary Figure 2), suggesting that a miR-24-3p low expression may be caused by lncRNA-FNIP2. The target relationship between lncRNA-FNIP2 and miR-24-3p means that lncRNA-FNIP2 not only affects the expression of *FNIP2* through cis regulation, but also by the lncRNA-FNIP2/miR-24-3p/*FNIP2* axis.

### *FNIP2* Promotes Preadipocyte Lipid Synthesis

**FIGURE 9:**
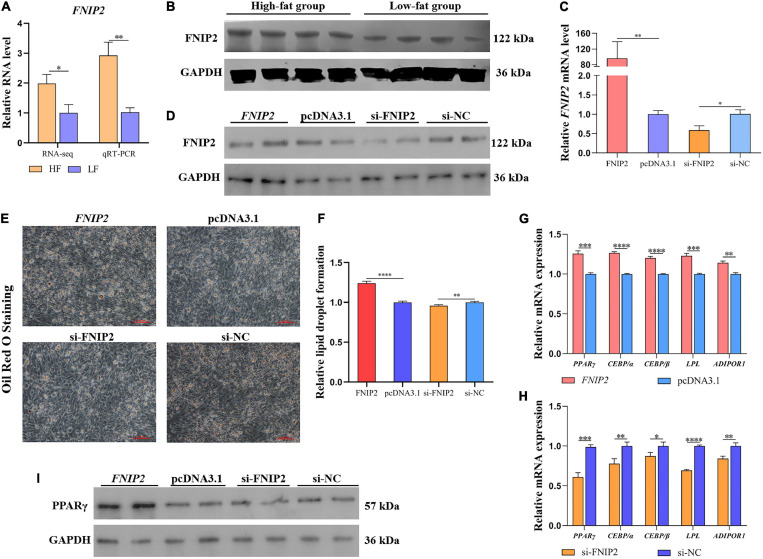
*FNIP2* promotes preadipocyte lipid synthesis. **(A)** The mRNA expression difference of *FNIP2* in liver between high-fat group and low-fat group was validated by qRT-PCR. **(B)** The protein expression difference of FNIP2 in liver between the high-fat and low-fat groups was validated by Western Blot. **(C,D)** The transfection efficiency of *FNIP2* overexpressed plasmid and si-FNIP2 in ICP1 was verified by qRT-PCR and Western Blot. **(E,F)** The lipid droplet formation in ICP1 was detected by Oil Red O Staining. **(G,H)** Relative mRNA expression of *PPAR*γ, *CEBP/*α, *CEBP/*β, *LPL*, and *ADIPOR1* in ICP1 was quantified by qRT-PCR. **(I)** The PPARγ protein in ICP1 was detected by Western Blot. ^∗^*P* < 0.05; ^∗∗^*P* < 0.01; ^∗∗∗^*P* < 0.001; ^****^P < 0.0001; ns, no significance.

In order to validate the expression difference of *FNIP2* between high-fat and low-fat groups, qRT-PCR was performed and the result showed that the *FNIP2* RNA level in the high-fat group was higher than that in the low-fat group (Figure 9A). In addition, the same trend was observed in the FNIP2 protein level (Figure 9B). The results indicate that FNIP2 was high-expressed in the liver of high-fat individuals. To verify the potential role of *FNIP2* in adipogenesis, we constructed the overexpression plasmid and synthesized the specific siRNA of *FNIP2*. The transfection efficiency of the plasmid and siRNA in ICP1 cells was validated both in the mRNA and protein levels (Figures 9C,D). ICP1 cells were induced to differentiate and the lipid droplet formation was detected by Oil Red O Staining. It could be found that *FNIP2* was able to induce more lipid droplet formation, while the knockdown of *FNIP2* could suppress lipid droplet formation in the ICP1 cells (Figures 9E,F). *FNIP2* could upregulate the mRNA expression of some lipid biosynthesis-related genes (including *PPAR*γ, *CEBP/*α, *CEBP/*β, *LPL*, and *ADIPOR1*), while the knockdown of *FNIP2* downregulated their mRNA expression (Figures 9G,H). Besides, PPARγ protein level was increased by *FNIP2*, indicating that PPARγ signal could be intensified by FNIP2 (Figure 9I). In short, these results suggest that the upregulated *FNIP2* in high-fat individuals could facilitate preadipocytes lipid synthesis.

### miR-24-3p Suppresses Lipid Synthesis by Targeting to *FNIP2*

**FIGURE 10:**
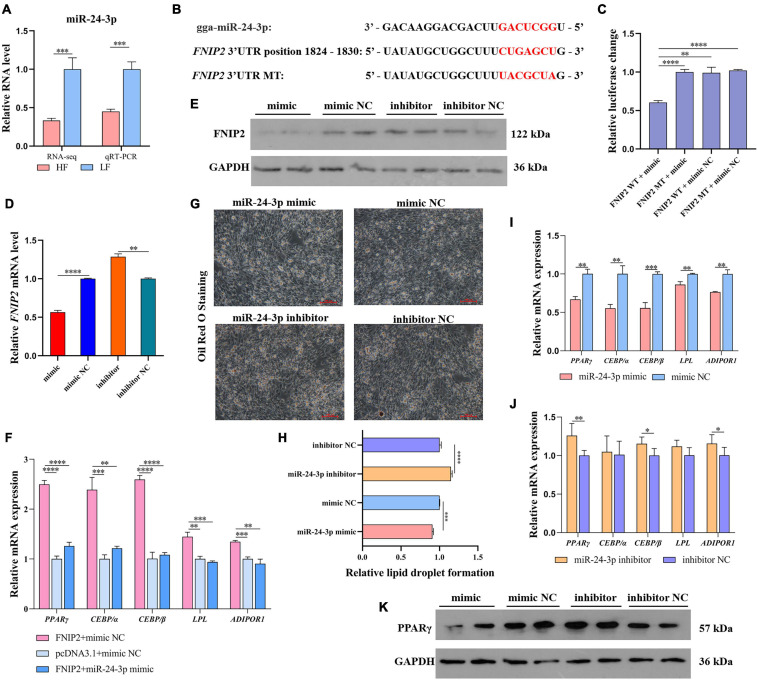
miR-24-3p suppresses lipid synthesis by targeting to *FNIP2*. **(A)** The RNA level difference of miR-24-3p in liver between the high-fat and low-fat groups was validated by qRT-PCR. **(B)** The target prediction between miR-24-3p and *FNIP2*. **(C)** The target relationship between miR-24-3p and *FNIP2* was validated by dual-luciferase reporter assay. **(D,E)** The mRNA and protein expression of FNIP2 in ICP1 was detected by qRT-PCR and Western Blot. **(F)** The restored effect of miR-24-3p on the mRNA expression of *PPAR*γ, *CEBP/*α, *CEBP/*β, *LPL*, and *ADIPOR1* in ICP1 was verified by qRT-PCR. **(G,H)** The lipid droplet formation in ICP1 was detected by Oil Red O Staining. **(I,J)** The mRNA expression of *PPAR*γ, *CEBP/*α, *CEBP/*β, *LPL*, and *ADIPOR1* in ICP1 was quantified by qRT-PCR. **(K)** The PPARγ protein in ICP1 was detected by Western Blot. ^∗^*P* < 0.05; ^∗∗^*P* < 0.01; ^∗∗∗^*P* < 0.001; ^****^P < 0.0001; ns, no significance.

The expression difference of miR-24-3p was detected by qRT-PCR and the expression trend was consistent with the sequencing result (Figure 10A). Based on the target prediction above between miR-24-3p and *FNIP2* (Figure 10B), we carried out a dual-luciferase reporter assay to verify the binding between miR-24-3p and *FNIP2*. It was showed that the co-transfection between wild type *FNIP2* 3′UTR and miR-24-3p could reduce the luciferase activity (Figure 10C), indicating a target relationship between *FNIP2* and miR-24-3p. qRT-PCR and the Western Blot results showed that miR-24-3p does have a posttranscriptional regulation on *FNIP2*, which miR-24-3p downregulated *FNIP2* not only at the mRNA level but also at the protein level (Figures 10D,E). Considering their target relationship, we performed another co-transfection to verify whether the promotion of *FNIP2* on lipid synthesis can be modulated by miR-24-3p. We found that the upregulation of *PPAR*γ, *CEBP/*α, *CEBP/*β, *LPL*, and *ADIPOR1* caused from *FNIP2* could be restored to normal levels by miR-24-3p (Figure 10F), being in accord with our expectation. In the Oil Red O Staining, it was found that miR-24-3p expression could decrease lipid droplet formation (Figures 10G,H). In addition, miR-24-3p was able to downregulate *PPAR*γ, *CEBP/*α, *CEBP/*β, *LPL*, and *ADIPOR1*, while miR-24-3p inhibitor upregulated their expression (Figures 10I,J). With the expression of miR-24-3p, PPARγ protein level was reduced, which manifested an inhibition of the miR-24-3p on the PPARγ signal (Figure 10K). These results suggest that miR-24-3p may cause lipid synthesis inhibition by targeting and downregulating *FNIP2*.

### *LncRNA*-FNIP2 Accelerates Lipid Synthesis by Releasing *FNIP2* From miR-24-3p

**FIGURE 11:**
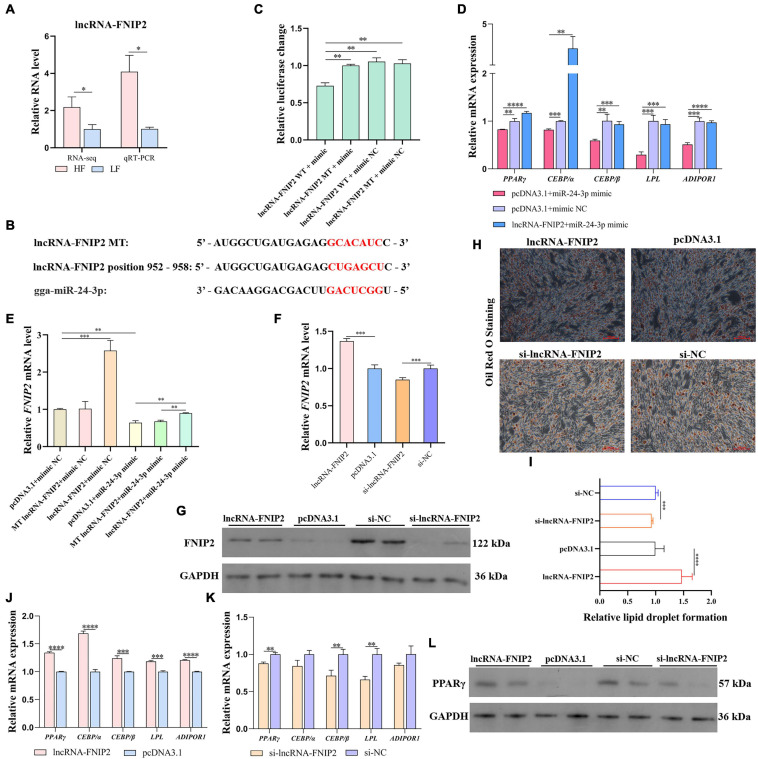
LncRNA-FNIP2 accelerates lipid synthesis by releasing *FNIP2* from miR-24-3p. **(A)** The RNA level difference of lncRNA-FNIP2 in liver between high-fat group and low-fat group was validated by qRT-PCR. **(B)** The target prediction between miR-24-3p and lncRNA-FNIP2. **(C)** The target relationship between miR-24-3p and lncRNA-FNIP2 was validated by dual-luciferase reporter assay. **(D)** The restore effect of lncRNA-FNIP2 on the mRNA expression of *PPAR*γ, *CEBP/*α, *CEBP/*β, *LPL*, and *ADIPOR1* in ICP1 was verified by qRT-PCR. **(E)** The restore effect of lncRNA-FNIP2 on *FNIP2* mRNA expression in ICP1 was verified by qRT-PCR. **(F,G)** The mRNA and protein expression of FNIP2 in ICP1 was detected by qRT-PCR and Western Blot. **(H,I)** The lipid droplet formation in ICP1 was detected by Oil Red O Staining. **(J,K)** The mRNA expression of *PPAR*γ, *CEBP/*α, *CEBP/*β, *LPL*, and *ADIPOR1* in ICP1 was quantified by qRT-PCR. **(L)** The PPARγ protein in ICP1 was detected by Western Blot. ^∗^*P* < 0.05; ^∗∗^*P* < 0.01; ^∗∗∗^*P* < 0.001; ^****^P < 0.0001; ns, no significance.

The expression difference of lncRNA-FNIP2 between the high-fat and low-fat groups was validated by qRT-PCR (Figure 11A). The above results indicated that lncRNA-FNIP2 may affect *FNIP2* through ceRNA mechanism (by miR-24-3p) during adipogenesis, and lncRNA-FNIP2 was predicted to adsorb miR-24-3p at positions 952–958 (Figure 11B). To verify the potential target relationship between lncRNA-FNIP2 and miR-24-3p, we performed a dual-luciferase reporter assay. It could be found that the luciferase activity in the co-transfection group of wild type lncRNA-FNIP2 and miR-24-3p was significantly lower than the other groups (Figure 11C), revealing their target relationship. In restored validation, we found that the mRNA expression of *PPAR*γ, *CEBP/*α, *CEBP/*β, *LPL*, and *ADIPOR1*, which have been reduced by miR-24-3p, could be restored or reversed by lncRNA-FNIP2 overexpression (Figure 11D). Based on the situation that both lncRNA-FNIP2 and *FNIP2* have a target relationship with miR-24-3p, we tried to verify whether lncRNA-FNIP2 could release *FNIP2* from miR-24-3p. It could be found that lncRNA-FNIP2 was be able to restore the *FNIP2* mRNA level from miR-24-3p, while the mutation of miR-24-3p binding site on lncRNA-FNIP2 lost this modulation function (Figure 11E). Subsequently, lncRNA-FNIP2 also showed a promoting effect on FNIP2 both at the mRNA and protein levels (Figures 11F,G). Similarly, Oil Red O staining indicated an effect of lncRNA-FNIP2 on accelerating lipid droplet formation (Figures 11H,I). Additionally, lncRNA-FNIP2 increased the mRNA levels of *PPAR*γ, *CEBP/*α, *CEBP/*β, *LPL*, and *ADIPOR1*, while lncRNA-FNIP2 knockdown decreased them (Figures 11J,K). LncRNA-FNIP2 could facilitate PPARγ protein expression, suggesting a promoting effect of lncRNA-FNIP2 on the PPARγ signal. These results reveal a positive role of lncRNA-FNIP2 on lipid synthesis, mediated by lncRNA-FNIP2/miR-24-3p/*FNIP2* axis during the adipogenesis process.

## Discussion

Lipid synthesis is a crucial process in animal fat accumulation and epigenetics has been characterized in vertebrate lipid metabolism and lipogenesis (Fu et al., 2015; Gao et al., 2016; Hahn et al., 2017). Recently, lncRNAs have been identified to be associated with adipogenesis (Xiao et al., 2015; Li et al., 2016). However, the regulatory role of lncRNA on chicken abdominal fat deposition is still unclear. In this study, we tried to gain an insight of the regulatory mechanism on chicken abdominal fat deposition by performing a high throughput sequencing analysis at different levels (including lncRNA, mRNA, and miRNA). A total of 2,265 DELs, 5,315 DEMs, and 245 DESs were obtained from our sequencing analysis. We constructed two interaction networks based on the cis regulation relationship between 68 DEGs and 97 DELs. Besides, the interaction network between 19 DEGs and eight DESs was constructed. Based on these interaction networks, the potential target axis lncRNA-FNIP2/miR-24-3p/*FNIP2* was hypothesized as a candidate regulatory mechanism in chicken adipogenesis.

Cross-talk between adjacent genes is a common phenomenon, involving multiple regulatory mechanisms and cis-regulatory signals (Engreitz et al., 2016). Recent researches suggested that the activity of transcription or DNA elements in lncRNA locus may be the way for lncRNA to regulate adjacent genes in cis (Kopp and Mendell, 2018). The potential of lncRNA during chicken abdominal fat adipocytes differentiation has been characterized (Zhang et al., 2017b,2020b). In this research, we constructed the cis-regulated networks between DEL and DEG. The potential cis-regulation relationships of five up-regulated lncRNAs and five down-regulated lncRNAs with their adjacent genes were respectively speculated, including lncRNA-GBE1/*GBE1*, lncRNA-PEX5L/*PEX5L*, lncRNA-PARD3/*PARD3*, lncRNA-NTNG1/*NTNG1*, lncRNA-FNIP2/*FNIP2*, lncRNA-SPIA3/*SPIA3*, lncRNA-SLC38A2/*SLC38A2*, lncRNA-IGF1/*IGF1*, lncRNA-JPH2/*JPH2*, and lncRNA-SOX7/*SOX7*. GBE1 (glycogen-branching enzyme 1) is required in glycogen accumulation for maintaining glucose metabolism balance (Thon et al., 1993; Bao et al., 1996; Froese et al., 2015). Our previous study found that *GBE1* was upregulated in the liver from the fast-growing WRR chickens (Claire et al., 2013). Recently, *GBE1* was suggested to be associated with backfat thickness traits (Ma et al., 2019), implying that lncRNA-GBE1 may participate in the glycogen metabolic process by regulating *GBE1* in cis. Parental high-fat diet increased offspring obesity and type 2 diabetes mellitus risks through the regulation of *SLC28A2* (solute carrier family 38 member 2) expression (Krout et al., 2018; Claycombe-Larson et al., 2020), which indicated that lncRNA-*SLC28A2* may be associated with chicken lipid synthesis.

In our previous research, *LPIN1*, downregulated by miR-429 in high-fat group abdominal fat tissues (the same individuals used in this study), inhibited lipid droplet formation. For epigenetics, miRNA play a pivotal role in the chicken lipid metabolic process (Chen et al., 2019; Sun et al., 2019; Zhang et al., 2020a). In this study, we also performed a small RNA sequencing and obtained 245 DESs. Compared with the sequencing data in abdominal fat tissues, relatively few miRNAs were detected in the liver and we did not observe miR-429-3p, indicating that it may be expressed ectopic in abdominal fat. Here, we hypothesized a potential mechanism that *LPIN1* expression abnormity may be caused by the transcriptional activity reduction of the upstream lncRNA-LPIN1 gene locus. We constructed a target network between DEMs and DESs, which was formed by 13 negative target pairs and 10 positive target pairs. Two genes, including *FNIP2* and *PEX5L*, are shared between the DESs-DEMs and DELs-DEMs networks. The cytosolic receptor, PEX5L (peroxisome biogenesis factor 5 long isoform), could recognize peroxisomal targeting signals 1 or 2 and peroxisomal import of matrix proteins would be initiated, which are involved in several metabolic processes (including lipid biosynthesis, fatty acid α-, and β-oxidation) (Hasan et al., 2013; Wanders, 2014). In our study, *PEX5L* was upregulated by the synergistic action of low expressed miR-15a and high expressed lncRNA-PEX5L, implying their potential on chicken liver lipid biosynthesis. Similar to miR-429-3p, miR-15a and miR-24-3p did not show a different expression trend in chicken abdominal fat tissues, which also characterized their tissue specificity. The reduction of miR-24-3p may be the reason for the increase of the fat mass and leptin levels during the treatment improvement of HD (Huntington’s disease) patients (Aganzo et al., 2018). In addition, miR-24-3p was also downregulated during the bovine preadipocytes differentiation process, which was predicted to target the *FASN* (fatty acid synthase) gene (Yu et al., 2020). Our research also revealed that miR-24-3p low expression in liver might cause more lipid formation.

The novel lncRNA-FNIP2 was predicted to sponge miR-24-3p and dual-luciferase reporter assay indeed identified their target relationship, as well as *FNIP2*. Here, we verified their role on lipid synthesis and it was found that lncRNA-FNIP2 and *FNIP2* were able to facilitate ICP1 lipid synthesis, while miR-24-3p inhibited lipid synthesis. Nutrient-rich conditions activate mTORC1 to trigger downstream anabolic reactions (Shen et al., 2019). FNIP2 (folliculin interacting proteins 2) could form a complex with FLCN. The complex would directly contact the Rag GTPases to stimulate GTP hydrolysis to GDP-bound state, which would promote mTORC1 activation (Tsun et al., 2013), indicating that FNIP2 may contribute to the mTORC1 signal to accelerate lipid synthesis, but further verification is needed to clarify the underlying mechanism. In this study, we performed a restored experiment and it showed that lncRNA-FNIP2 did release *FNIP2* from miR-24-3p, suggesting the objective reality of the lncRNA-FNIP2/miR-24-3p/*FNIP2* axis. Besides, the promotion of the lncRNA-FNIP2/miR-24-3p/*FNIP2* axis on the PPARγ signal was preliminary demonstrated, suggesting a positive effect of the lncRNA-FNIP2/miR-24-3p/*FNIP2* axis on lipid metabolism. In short, we screened the lncRNA-FNIP2/miR-24-3p/*FNIP2* axis that may be related to abdominal fat deposition through the whole transcriptome analysis, and verified the real existence of the lncRNA-FNIP2/miR-24-3p/*FNIP2* axis and its promoting effect on lipid synthesis.

## Data Availability Statement

The datasets presented in this study can be found in online repositories. The names of the repository/repositories and accession number(s) can be found below: https://www.ncbi.nlm.nih.gov/, PRJNA684949 and https://www.ncbi.nlm.nih.gov/, PRJNA686699.

## Ethics Statement

The animal study was reviewed and approved by the Animal Care Committee of South China Agricultural University. Written informed consent was obtained from the owners for the participation of their animals in this study.

## Author Contributions

LG was responsible for the research designing, data analysis, and manuscript writing. XC participated partly in the experiments and data analysis. WH and ZL reviewed and modified the manuscript. KL, MY, SZ, ML, HL, WL, and XZ were responsible for the animal experiments and sample collection. QN participated in the research designing. QL carried out the design of the whole research and guided the research progress. All authors contributed to the article and approved the submitted version.

## Conflict of Interest

The authors declare that the research was conducted in the absence of any commercial or financial relationships that could be construed as a potential conflict of interest.

## Footnotes

1 https://www.ncbi.nlm.nih.gov/bioproject/PRJNA684949

2 https://www.ncbi.nlm.nih.gov/bioproject/PRJNA686699

3 https://www.mdc-berlin.de/8551903/en/

4 http://www.bioconductor.org/packages/release/bioc/html/DEGseq.html

5 http://www.microrna.org/microrna/home.do

6 https://bibiserv.cebitec.uni-bielefeld.de/rnahybrid

## Supplementary Material

Supplementary Figure 1 The coding capacity of novel transcripts, distribution of RNA length, and isoforms expression in each sample.

Supplementary Figure 2 The target relationship prediction of miR-24-3p.

Supplementary Table 1 Fragment sequence information.

Supplementary Table 2 The information of sequencing data.

Supplementary Table 3 Genome alignment statistical of clean reads.

Supplementary Table 4 The number statistics of lncRNA and mRNA isoforms.

Supplementary Table 5 The differently expressed lncRNA between high-fat and low-fat groups.

Supplementary Table 6 The differently expressed genes between high-fat and low-fat groups.

Supplementary Table 7 The differently expressed mRNAs between high-fat and low-fat groups.

Supplementary Table 8 The GO term DEMs enriched in.

Supplementary Table 9 The KEGG pathway DEMs enriched in.

Supplementary Table 10 The statistics of DELs cis regulation.

Supplementary Table 11 Classification statistics of DEL overlap with mRNA.

Supplementary Table 12 The KEGG pathway differently expressed DELs’ target genes enriched in.

Supplementary Table 13 Target pairs between DELs and DEGs.

Supplementary Table 14 Small RNA sequencing data statistics.
