# Supplementary material for: Whole Transcriptome Analysis Reveals a Potential Regulatory Mechanism of LncRNA-FNIP2/miR-24-3p/FNIP2 Axis in Chicken Adipogenesis
Source: Front Cell Dev Biol. 2021 Jun 24;9:653798. doi: 10.3389/fcell.2021.653798 (PMC8265275; doi:10.3389/fcell.2021.653798)
Supplement: Supplementary Figure 1 — The coding capacity of novel transcripts, distribution of RNA length, and isoforms expression in each sample. [file Data_Sheet_1.zip › Figures, Tables 1-4, 10-11, 13-14.pdf]

Supplementary Figures:

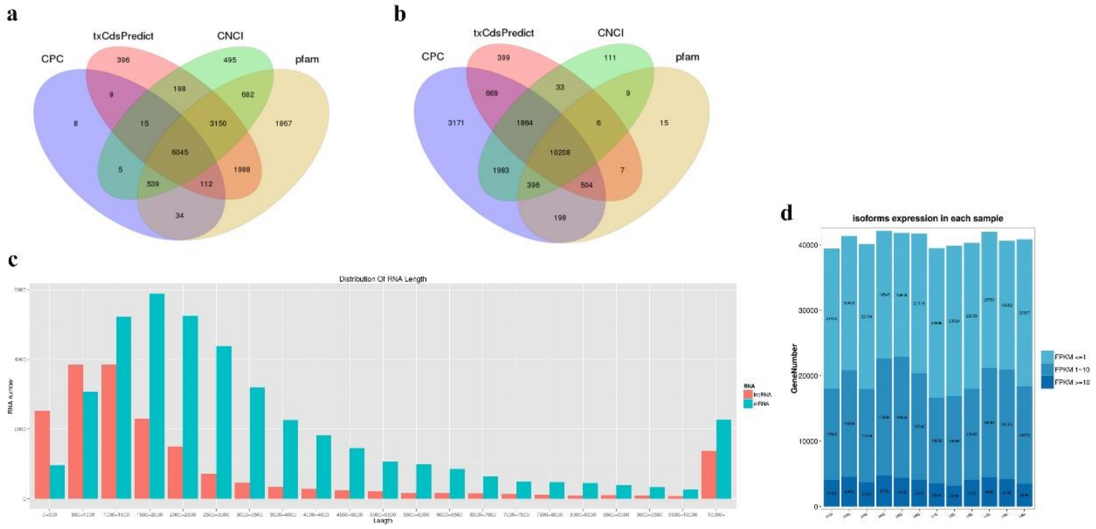

Figure. S1. The coding capacity of novel transcripts, the distribution of RNA length and isoforms expression in each sample. (a) 9,861 novel lncRNAs were identified by CPC, txCdsPredict, CNC and pfam. (b) 12,978 novel mRNAs were identified by CPC, txCdsPredict, CNC and pfam. (c) The distribution of RNA length. (d) The isoforms expression in each sample.

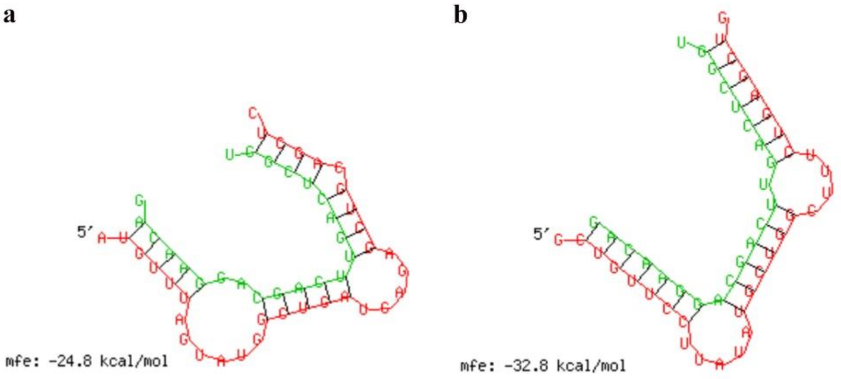

Figure. S2. The target relationship prediction of miR-24-3p. (a) The target prediction between lncRNA-FNIP2 and miR-24-3p. (b) The target prediction between *FNIP2* and miR-24-3p.

Supplementary Tables:

Supplementary Table 1. Fragment sequence information.

Fragment name	Fragment sequences (5' to 3')	Application
lncRNA-FNIP2 F	GCTTGGAGAGTGAATCAGAAGG	qRT-PCR
lncRNA-FNIP2 R	TTGGGAACAGAGGCAGTAGC	qRT-PCR
FNIP2 F	TTAACTGCGGTGCTGACGTA	qRT-PCR
FNIP2 R	GGCACCATAGAGATCACCAAGC	qRT-PCR
PPARγ F	TCCTTCCCGCTGACCAAA	qRT-PCR
PPARγ R	TCCTGCACTGCCTCCACA	qRT-PCR
LPL F	CCAAGGTAGACCAGCCATTC	qRT-PCR
LPL R	TGCTCCAGGCACTTCACA	qRT-PCR
ADIPOR1 F	GACAAGAACAGCAACGAGTACCGC	qRT-PCR
ADIPOR1 R	CCTGAAGATGCCCCGCAGAGT	qRT-PCR
CEBP/α F	GACAAGAACAGCAACGAGTACCGC	qRT-PCR
CEBP/α R	CCTGAAGATGCCCCGCAGAGT	qRT-PCR
CEBP/β F	GCGGACTGTTTGGCTGCTCT	qRT-PCR
CEBP/β R	CGGGTGAGGCTGATGTAGGTGT	qRT-PCR
GAPDH F	CAACTTTGGCATTGTGGAGG	qRT-PCR
GAPDH R	CGCTGGGATGATGTTCTGG	qRT-PCR
si-lncRNA-FNIP2	GCTATTCCACTGTAACATT	lncRNA-FNIP2 knockdown
si-FNIP2	CCTCTTTGCCATACAGTAA	FNIP2 knockdown
gga-miR-24-3p mimic	UGGCUCAGUUCAGCAGGAACAG	gga-miR-24-3p overexpression
gga-miR-24-3p inhibitor	ACCGAGUCAAGUCGUCCUUGUC	gga-miR-24-3p knockdown

Supplementary Table 2. The information of sequencing data.

Sample	Total Raw Reads	Total Clean Reads	Total Clean Base	GC	Q30	Clean Reads Ratio
H1G	126,145,598	126,145,598	12,614,559,800	43.42%	93.92%	100.000%
H2G	126,252,822	126,252,822	12,625,282,200	44.42%	93.78%	100.000%
H3G	126,980,494	126,980,494	12,698,049,400	44.37%	93.64%	100.000%
H4G	134,931,636	127,737,134	12,773,713,400	49.37%	92.46%	94.668%
H5G	115,457,126	110,224,456	11,022,445,600	50.35%	93.19%	95.468%
H6G	134,931,636	126,636,920	12,663,692,000	50.83%	92.49%	93.853%
L1G	126,824,198	126,824,198	12,682,419,800	44.64%	93.53%	100.000%
L2G	126,956,754	126,956,754	12,695,675,400	44.3%	93.54%	100.000%
L3G	127,003,910	127,003,910	12,700,391,000	44.54%	93.89%	100.000%
L4G	132,432,902	126,207,230	12,620,723,000	49.58%	92.54%	95.299%
L5G	132,432,902	126,127,716	12,612,771,600	50.33%	92.74%	95.239%
L6G	132,432,902	128,192,818	12,819,281,800	48.46%	92.41%	96.798%

Supplementary Table 3. Genome alignment statistical of clean reads.

Sample	Total Mapping Clean Reads	Total Mapping Ratio	Uniquely Mapping Ratio
H1G	101,295,252	92.26%	91.08%
H2G	106,737,260	91.62%	90.34%
H3G	100,431,050	91.86%	90.39%
H4G	109,699,062	86.97%	84.26%
H5G	91,750,362	86.92%	83.66%
H6G	119,407,594	86.27%	83.17%
L1G	98,228,418	91.90%	90.32%
L2G	98,497,344	92.01%	90.51%
L3G	100,916,940	92.39%	90.80%
L4G	119,986,694	87.28%	84.27%
L5G	103,580,312	86.17%	83.08%
L6G	102,292,692	89.15%	86.62%

Supplementary Table 4. The number statistics of lncRNA isoforms and mRNA isoforms.

Sample	Novel lncRNA isoforms	Novel mRNA isoforms	Known lncRNA isoforms	Known mRNA isoforms
L1G	8005	10350	2240	18921
L6G	8651	10607	2171	19437
L4G	8794	10721	2529	19964
L3G	8168	10414	2419	19328
H3G	8330	10438	2410	18975
H5G	8944	10650	2484	19742
H6G	8801	10739	2365	19823
H4G	8850	10756	2574	19991
H1G	8144	10246	2422	18643
L2G	8311	10380	2386	18822
L5G	8841	10546	2289	18955
H2G	8461	10607	2674	19641

Supplementary Table 10. The statistics of DELs cis regulation.

Region	TarPair Number	mRNA Number	lncRNA Number	lncRNA location (upstream/downstream)
overlap	2605	1984	1717	-
1-1000	796	733	630	upstream
1001-2000	348	309	273	upstream
2001-3000	243	228	201	upstream
3001-4000	217	204	175	upstream

4001-5000	211	195	184	upstream
5001-6000	169	152	134	upstream
6001-7000	185	173	154	upstream
7001-8000	168	161	140	upstream
8001-9000	137	127	118	upstream
9001-10000	159	150	140	upstream
10001-11000	1	1	1	upstream
1-1000	513	468	381	downstream
1001-2000	302	286	254	downstream
2001-3000	411	392	337	downstream
3001-4000	319	292	256	downstream
4001-5000	271	257	223	downstream
5001-6000	220	214	177	downstream
6001-7000	199	183	170	downstream
7001-8000	183	173	140	downstream
8001-9000	155	143	134	downstream
9001-10000	148	137	125	downstream
10001-11000	139	122	117	downstream
11001-12000	152	140	128	downstream
12001-13000	153	142	123	downstream
13001-14000	120	116	101	downstream
14001-15000	145	139	126	downstream
15001-16000	128	123	104	downstream
16001-17000	112	107	95	downstream
17001-18000	125	117	98	downstream
18001-19000	117	114	97	downstream
19001-20000	91	87	78	downstream

Supplementary Table 11. Classification statistics of DEL overlap with mRNA.

Overlap Class	lncRNA Number	mRNA Number	Pair Number
Lnc-Overlap-mRNA	628	774	907
Lnc-AntiOverlap-mRNA	591	724	833
Lnc-CompleteIn-mRNAExon	17	18	20
Lnc-AntiCompleteIn-mRNAExon	39	44	45
mRNA-CompleteIn-LncExon	9	9	9
mRNA-AntiCompleteIn-LncExon	1	1	1
Lnc-CompleteIn-mRNAIntron	428	395	569
Lnc-AntiCompleteIn-mRNAIntron	77	78	94
mRNA-CompleteIn-LncIntron	13	13	14
mRNA-AntiCompleteIn-LncIntron	17	19	20

Supplementary Table 13. Target pairs between DELs and DEGs.

lncRNA ID	Gene name	lncRNA ID	Gene name	lncRNA ID	Gene name
LTCONS_00050642	*SPIA3*	LTCONS_00051969	*SORCS3*	LTCONS_00045938	*ETNPPL*

LTCONS_00050643	*SPIA3*	LTCONS_00027368	*LOC100859272*	LTCONS_00027367	*LOC100859272*
LTCONS_00002483	*GBE1*	LTCONS_00042745	*SLC6A14*	LTCONS_00044689	*POLN*
LTCONS_00011548	*MAPKAPK3*	LTCONS_00038239	*FOSL2*	LTCONS_00014228	*EGR1*
LTCONS_00058214	*PEX5L*	ENSGALT00000091473	*SEC31B*	LTCONS_00039584	*SH3YL1*
LTCONS_00037430	*SOX7*	LTCONS_00026243	*PKIA*	LTCONS_00044688	*POLN*
LTCONS_00011301	*CDH13*	LTCONS_00049587	*PTPN5*	LTCONS_00052907	*IFIT5*
LTCONS_00021300	*PARD3*	LTCONS_00000615	*SLC38A2*	LTCONS_00052961	*KIF11*
LTCONS_00001244	*IGF1*	LTCONS_00049580	*PTPN5*	LTCONS_00027360	*LOC100859272*
LTCONS_00039704	*LPIN1*	LTCONS_00054242	*MYLK*	ENSGALT00000096614	*LOC100859272*
LTCONS_00005023	*SLC38A2*	LTCONS_00000620	*SLC38A4*	LTCONS_00012216	*RASSF1*
LTCONS_00062791	*HMGCS1*	LTCONS_00052849	*MSMB*	LTCONS_00031015	*POU2AF1*
LTCONS_00056507	*NTNG1*	LTCONS_00037429	*SOX7*	LTCONS_00033273	*PLXDC1*
LTCONS_00043809	*AREG*	LTCONS_00013788	*CSNK1A1*	LTCONS_00044687	*POLN*
LTCONS_00043262	*FNIP2*	LTCONS_00060416	*JPH2*	ENSGALT00000094571	*MAPKAPK3*
LTCONS_00031018	*POU2AF1*	ENSGALT00000086781	*LOC100859272*	LTCONS_00033537	*PLCD3*
LTCONS_00020764	*NUFIP2*	ENSGALT00000062963	*LOC101750289*	LTCONS_00058829	*PLSCR1*
LTCONS_00059689	*LOC100859100*	LTCONS_00043519	*SH2D4A*	LTCONS_00057780	*STK25*
LTCONS_00000899	*HAL*	LTCONS_00025492	*LOC112530660*	LTCONS_00047058	*WHSC1*
LTCONS_00019476	*SMIM5*	LTCONS_00025492	*LOC100857299*	LTCONS_00010433	*TDRD12*
LTCONS_00060413	*JPH2*	LTCONS_00025492	*LOC112531552*	LTCONS_00060505	*JPH2*
LTCONS_00021304	*PARD3*	LTCONS_00023947	*GJC2*	ENSGALT00000094930	*NUBPL*
LTCONS_00006662	*GBE1*	LTCONS_00014734	*GRIN2A*	LTCONS_00055090	*ITGB6*
LTCONS_00048252	*SPTBN5*	LTCONS_00000900	*HAL*	ENSGALT00000097244	*LOC100857860*
LTCONS_00062792	*HMGCS1*	LTCONS_00026242	*PKIA*	ENSGALT00000102508	*GALNT3*
LTCONS_00017309	*BG8*	ENSGALT00000031441	*TK1*	LTCONS_00025671	*IFI6*
LTCONS_00005024	*SLC38A2*	LTCONS_00031017	*POU2AF1*	LTCONS_00049581	*PTPN5*
ENSGALT00000105182	*HMGCS1*	LTCONS_00063021	*KANK1*	ENSGALT00000091646	*LOC101750289*
LTCONS_00054243	*MYLK*	LTCONS_00006617	*MAEL*	LTCONS_00048584	*RPS6KL1*
LTCONS_00037428	*SOX7*	LTCONS_00017301	*BG8*	ENSGALT00000090652	*DPP10*
LTCONS_00054367	*KYNU*	LTCONS_00037637	*CD248*	LTCONS_00048763	*SERPINA10*
LTCONS_00061290	*HMGCS1*	LTCONS_00049588	*PTPN5*	LTCONS_00060415	*JPH2*
LTCONS_00007222	*STS*	LTCONS_00031016	*POU2AF1*	LTCONS_00047583	*LOC107053122*
LTCONS_00005826	*USP41*	LTCONS_00005825	*USP41*	LTCONS_00033691	*SRCIN1*

Supplementary Table 14. Small RNA sequencing data statistics.

Sample name	Raw tag count	Clean tag count	Q20 of clean tag (%)	Percentage of clean tag (%)	Mapped tag	Percentage of mapping (%)
H1G	24201194	22439805	99.1	92.72	19724932	84.69
H2G	23570600	22179633	98.3	94.1	19536395	88.08
H3G	25130241	22416852	99	89.2	19757641	88.14
H4G	25020312	22942107	99.5	91.69	20483262	89.28
H5G	23264946	21474128	99.4	92.3	18977263	88.37
H6G	24282500	22497733	99.4	92.65	20245310	89.99
L1G	25038255	22042843	99.1	88.04	19571308	88.79
L2G	24247719	20349525	99.1	83.92	17018886	83.63
L3G	23666595	20254713	99.1	85.58	19090088	94.25

L4G	24279297	21984813	99.3	90.55	19750863	89.84
L5G	24181905	20972660	99.4	86.73	19298391	92.02
L6G	24034524	21154182	99.4	88.02	19725379	93.25
